# High OX40 expression in recurrent ovarian carcinoma is indicative for response to repeated chemotherapy

**DOI:** 10.1186/s12885-018-4339-0

**Published:** 2018-04-16

**Authors:** Michaela Ramser, Simone Eichelberger, Silvio Däster, Benjamin Weixler, Marko Kraljević, Robert Mechera, Athanasios Tampakis, Tarik Delko, Uwe Güth, Sylvia Stadlmann, Luigi Terracciano, Raoul A. Droeser, Gad Singer

**Affiliations:** 1 Department of Surgery, University Hospital Basel, Spitalstrasse 21, 4031 Basel, Switzerland; 2 Spital Limmattal, Urdorferstrasse 100, 8952 Schlieren, Switzerland; 3 Brustzentrum Zürich, Seefeldstrasse 214, 8008 Zürich, Switzerland; 4 Department of Gynecology and Obstetrics, University Hospital Basel, Spitalstrasse 21, 4031 Basel, Switzerland; 5 Institute of Pathology, Kantonsspital Baden AG, Im Ergel 1, 5404 Baden, Switzerland; 6 Institute of Pathology, University Hospital Basel, Schönbeinstrasse 40, 4031 Basel, Switzerland

**Keywords:** OX40, CD134, Ovarian cancer, Chemosensitivity

## Abstract

**Background:**

Ovarian carcinoma (OC) is the fifth most common female cancer and mostly diagnosed at an advanced stage. Surgical debulking is usually followed by adjuvant platinum-based chemotherapy. Only few biomarkers are known to be related to chemosensitivity. OX40 is a TNF receptor member and expressed on activated CD4+ and CD8+ T cells. It is known that OX40 signaling promotes survival and responds to various immune cells of the innate and adaptive immune system. Therefore we investigated the indicative value of OX40 expression for recurrence and survival in OC.

**Methods:**

A tissue microarray of biopsies of mostly high-grade primary serous OC and matched recurrences of 47 patients was stained with OX40. Recurrence within 6 months of the completion of platinum-based chemotherapy was defined as chemoresistance.

**Results:**

Chemosensitivity correlated significantly with high OX40 positive immune cell density in primary cancer biopsies (*p =* 0.027). Furthermore patients with a higher OX40 expression in recurrent cancer biopsies showed a better outcome in recurrence free survival (RFS) (*p =* 0.017) and high OX40 expression was associated with chemosensitivity (*p =* 0.008). OX40 positive TICI in recurrent carcinomas significantly correlated with IL-17 positive tumor infiltrating immune cells in primary carcinomas (*r*_*s*_ = 0.34; *p* = 0.023). Univariate cox regression analysis revealed a significant longer RFS and higher numbers of chemotherapy cycles for high OX40 tumor cell expression in recurrent cancer biopsies (HR 0.39, 95%CI 0.16–0.94, *p = 0.036* and 1.28, 95%CI 1.05–1.55; *p = 0.013*).

**Conclusion:**

High OX40 expression in OC is correlated with chemosensitivity and improved RFS in OC. Patients might therefore benefit from a second line therapy.

## Background

Ovarian carcinoma (OC) is the fifth most common cause of all cancer related deaths in women and has an incidence of 5–15/100′000 in Europe [1–3]. Often it is diagnosed at a late disease stage [4] and treated with surgical debulking and adjuvant chemotherapy. Of the various subtypes of OC, high-grade serous carcinoma is the most common subtype [5]. After surgical debulking, adjuvant platinum-based chemotherapy is currently the standard treatment modality.

It is known that tumor response to cytotoxic drugs shows great variability. The availability of predictive biomarkers for chemosensitivity of a given OC would be helpful to plan an individually adequate therapy. Such biomarkers would allow an individualized therapy with either repetitive chemotherapy cycles or extended surgical procedures or palliative care in cases of chemoresistance. Previous studies investigated several biomarkers related to chemosensitivity of platinum-based chemotherapy in OC, but only few helpful markers have so far been found [6–8].

It is widely known that tumor microenvironment influences tumor biology and that tumor behavior is affected by the immunological environment. Based on this background, a variety of different cancers with strong lymphatic infiltration have been investigated [9]. Especially in OC it has been shown that an increased number of intratumoral T cells correlates with an improved clinical outcome [9, 10]. Furthermore, in metastatic colorectal cancer the response to chemotherapy is associated with a high T cell density [11].

OX40 (CD134) is a co-stimulatory trans-membrane molecule and belongs to the tumor necrosis factor-receptor superfamily [12–14]. It is expressed on activated CD4+ and CD8+ T cells, as well as on other cell types [15–18]. The OX40 ligand, expressed by antigen-presenting cells (APC), activates the OX40 signaling pathway which promotes a robust immune response. The interaction of OX40 with OX40 ligand results in enhanced CD4+ and CD8+ cell proliferation, stimulated cytokine production, and increased survival of antigen-specific memory T cell [19–21]. In mice the absence of OX40 has been shown to cause a strong reduction in the number of effector memory CD4+ cells [22]. Furthermore, the CD8+ response was reduced and tumor growth was accelerated [19]. Accordingly, it is comprehensible that the immune-stimulating properties of OX40 agonists could overcome some of the immunosuppressive properties within tumor environment [23].

In a first clinical trial, OX40 targeted immunotherapy treatment has been tested in patients with different types of cancer and patients showed tumor regression after only one cycle of treatment. Furthermore, a significant dose-dependent increase in proliferation of CD4+ and CD8+ T cells was observed [24].

In a previous study by our group, we could demonstrate that infiltration with IL-17-positive tumor immune cell is indicative for an enhanced response to chemotherapy in primary and recurrent OC [25]. IL-17 has been shown to be produced by tumor-infiltrating lymphocytes as well as by granulocytes and other innate immune cells [26–28]. Additionally, in another study we were able to show that high density of myeloperoxidase (MPO) positive cell enhances the indicative value of IL-17 for response to chemotherapy in ovarian carcinoma [29].

In the future, adjuvant chemotherapy of patients with OC should be improved by an adapted regimen according to predictive markers on chemoresistance and –sensitivity. In the present study we investigated whether the analysis of OX40 could add significant value to available biomarkers and help predict chemosensitivity. The aim of this study was to address the predictive value of OX40 in OC. Our approach was based on the assumption that antitumor activity of chemotherapy regimen is partially based on the interaction between tumor cells and the immune system in a complex process [30].

## Methods

### Patients

One or two (in one third of the patients) tissueblocks from the resected high-grade serous OC and their recurrences from 47 patients were collected from the Institutes of Pathology of the University Hospital of Basel and the Cantonal Hospitals of Baden, Liestal, and St. Gallen, Switzerland (5.7% FIGO stage II, 84.3% FIGO stage III, and 4.3% FIGO stage IV) [31, 32]. All patients had initial surgical debulking, followed by at least three cycles of platinum-based adjuvant chemotherapy. All patients developed recurrences after initial surgery. In terms of response to chemotherapy, women were divided into a chemosensitive and a chemoresistant group according to the date of recurrence. Recurrence that occurred within 6 months after completion of platinum-based chemotherapy was defined as chemoresistant disease while recurrence diagnosed at a later state was defined as chemosensitive [33].

The statement concerning the clinical data collection and ethical considerations can be found in our previous publications [25, 34–37].

### Tissue microarray

The construction of the tissue microarray has been previously described. Briefly, formalin-fixed, paraffin-embedded tissue blocks of the resected tumor were prepared to construct the TMA. A representative tissue region of the hematoxylin-eosin stained tumor was selected and tissue cylinders of 0.6 mm in diameter were punched and sections of 3 μm transferred to the glass slide were they were available for immunohistochemial stainings [25, 38].

### Immunohistochemistry (IHC) and visual analysis

**Fig. 1:**
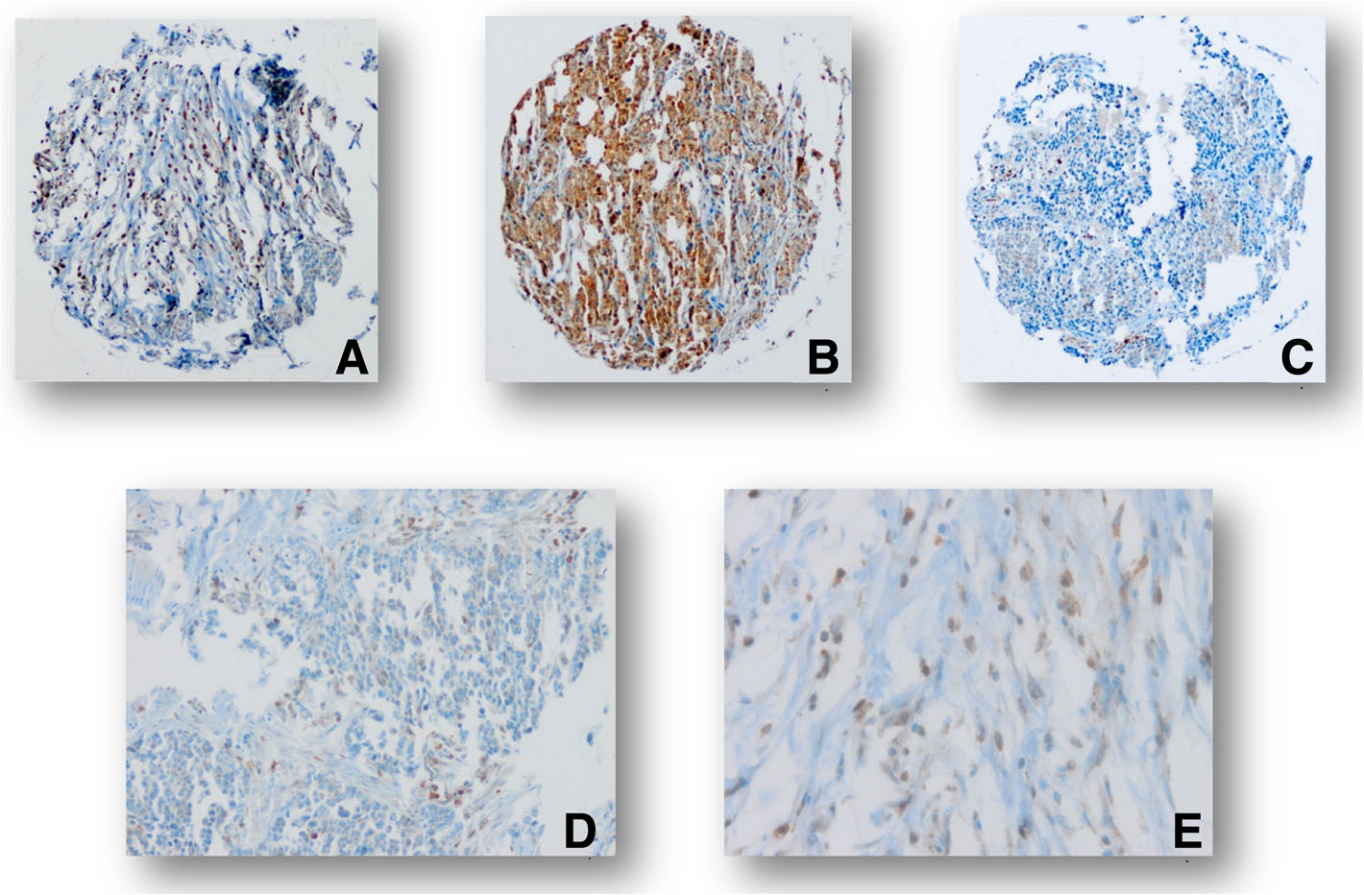
Example of high OX40 immune cell density (**a**), high OX40 tumor expression (**b**) and low immune cell density and low OX40 tumor expression (**c**) (10×). Positive immune cells (D (20×), E (40×)).

Standard indirect immunoperoxidase procedures (ABC-Elite, Vectra Laboratories) were used for immunohistochemistry. The TMA was stained for OX40 (clone Abcam ab119904) and each tissue spot was assessed twice. First, in each biopsy all positively stained tumor infiltrating immune cells (TICI) were counted. Second, in each biopsy only OX40-positive tumor cells were counted. Fibroblasts and macrophages were not counted. The assessment included the whole section plane of all biopsies. Intravascular located immune cells were excluded from analysis (Fig. 1). Two examiners (MR and SE) analysed the staining independently. The number of OX40 positive immune cells per punch were allocated to a high or low expression group (cut-off in primary carcinoma = 36.5 and cut-off in recurrent carcinoma = 2.5). For OX40 tumor cell expression the corresponding cut-off values were 144.5 for primary and 12.5 for recurrent carcinoma. Biopsies with less than 25% of morphologically preserved tissue were excluded from analysis. Conclusive data for OX40 was available in 47 biopsies of primary and 44 biopsies of matched recurrent carcinomas.

### Statistical analysis section

The assumption of proportional hazards was verified for the marker by analyzing the correlation of Schoenfeld residuals and the ranks of individual failure times. Any missing clinicopathological information was assumed to be missing at random. Subsequently, a multivariate Cox regression analysis was performed. The hazard ratios (HR) and the 95% confidence intervals (CI) were used to determine prognostic effects on survival time. Spearman’s rank correlation was used to analyze the correlation between OX40 tumor expression, OX40 immune cell density and IL-17. All statistical analyses were made using STATA software version 13 (StataCorp, College Station, TX, USA).

Cut-off scores to classify OC with low or high OX40 infiltration/expression were obtained by regression tree analysis, evaluating the best threshold in order to predict patients’ survival status, on all tumor samples [39]. IL-17 data were available from our previous publication [25, 29]. Kruskal Wallis, Chi-Square or Fisher’s Exact tests were used for the association of the clinicopathological features with the corresponding four groups of the biomarkers. Univariate recurrence-free and overall survival analysis was performed by the Kaplan-Meier method and log rank test.

## Results

### Patient characteristics

Median age of the cohort was 58.5 years (range 34–77). Thirty-eight patients (80.9%) had FIGO-stage III cancer. 70.2% (*n* = 33) were chemosensitive and 29.8% (*n* = 14) chemoresistant. The overall 6-month recurrence free survival (RFS) rate was 0.53 (0.38–0.66) and the corresponding 3-year overall survival (OS) rate was 0.47 (0.29–0.63) (Table 1). Expectedly, RFS and OS were significantly shorter for the patients in the chemoresistant group compared to the patients in the chemosensitive group (RFS: 2.2 ± 0.3 vs 18.2 ± 2.0 months, *p* < 0.0001 and OS: 27 ± 5.3 vs. 49.6 ± 4.0 months, *p =* 0.0003, respectively).

**Table 1:** Patient characteristics (*n* = 47)^a^

	*N* = 47 (100%)
Age (median, range)	58 (34–77)
FIGO stage	
II	1 (2.1)
IIIA	1 (2.1)
IIIB	5 (10.6)
IIIC	32 (68.2)
IV	8 (17.0)
Residual disease	
None	16 (34.0)
< 2 cm	17 (36.2)
> 2 cm	13 (27.7)
Numbers of chemotherapy cycles	
< 6	7 (14.9)
6 or more	39 (83.0)
CS^b^	33 (70.2)
CR^b^	14 (29.8)
6-month RFS % (95%CI)^c^	0.53 (0.38–0.66)
3-year OS % (95%CI)^c^	0.47 (0.29–0.63)

^a^missing clinicopathological information was assumed to be missing at random

^b^*CS* chemosensitive, *CR* chemoresistant

^c^*RFS* recurrence-free survival, *OS* overall survival

### OX40 positive immune cell infiltration in primary and recurrent OC

The mean number of infiltrating OX40 positive immune cell in primary and recurrent cancer biopsies was 25.6 (±4.5 SE) and 25.6 (±8.5 SE), respectively.

We found a significant association between high numbers of OX40 positive tumor infiltrating immune cells and improved chemosensitivity in primary cancer biopsies (*p =* 0.027) (Table 2). On the other hand, no significant association of immune cell count and chemosensitivity or resistance was found in biopsies from recurrent carcinomas (Table 3).

**Table 2:** Patients’ characteristics according to dichotomized distribution of OX40 positive immune cells in primary cancer biopsies in the overall cohort (cut-off = 36.5 cells; *n* = 47)^a^

	OX40 high	OX40 low	*p*-value
	*n* = 14 (100%)	*n* = 33 (100%)	
Age (median, range)	57 (41–73)	59 (34–77)	0.464
FIGO stage			0.795
II	0	1 (3.0)	
IIIA	0	1 (3.0)	
IIIB	2 (14.3)	3 (9.1)	
IIIC	11 (78.6)	21 (63.6)	
IV	1 (14.1)	7 (21.2)	
Residual disease			0.243
None	7 (50.0)	9 (27.3)	
< 2 cm	5 (35.7)	12 (36.4)	
> 2 cm	2 (14.3)	11 (33.3)	
Numbers of chemotherapy cycles			0.057
< 6	0	7 (21.2)	
6 or more	14 (100.0)	25 (75.8)	
CS^b^	13 (93.0)	20 (60.6)	0.027
CR^b^	1 (14.1)	13 (39.4)	
6-month RFS % (95%CI)^c^	0.71 (0.41–0.88)	0.45 (0.28–0.61)	0.461
3-year OS % (95%CI)^c^	0.38 (0.10–0.67)	0.46 (0.26–0.64)	0.780

^a^percentages may not add to 100% due to missing values of defined variables, missing clinicopathological information was assumed to be missing at random. Variables are indicated as absolute numbers, %, median or range. Age, RFS and OS were evaluated using the Kaplan-Meier method. FIGO stage, residual disease, numbers of chemotherapy cycles and chemoresistance were analyzed using the Chi-Square or the Fisher’s Exact test

^b^*CS* chemosensitive, *CR* chemoresistant

^c^*RFS* recurrence-free survival, *OS* overall survival

**Table 3:** Patients’ characteristics according to dichotomized distribution of OX40 positive immune cells in recurrent cancer biopsies in the overall cohort (cut-off = 2.5 cells; *n* = 44)^a^

	OX40 high	OX40 low	*p*-value
	*n* = 28 (100%)	*n* = 16 (100%)	
Age (median, range)	56.5 (34–73)	63.5 (41–77)	0.329
FIGO stage			
II	1 (3.6)	0	0.821
IIIA	0	1 (6.3)	
IIIB	3 (10.7)	2 (12.5)	
IIIC	19 (67.9)	10 (62.5)	
IV	5 (17.9)	3 (18.8)	
Residual disease			
None	10 (35.7)	5 (31.3)	0.850
< 2 cm	11 (39.3)	5 (31.3)	
> 2 cm	7 (25.0)	5 (31.3)	
Numbers of chemotherapy cycles			
< 6	5 (17.9)	2 (12.5)	0.702
6 or more	23 (82.1)	13 (81.3)	
CS^b^	22 (78.6)	9 (56.3)	0.118
CR^b^	6 (21.4)	7 (43.8)	
6-month RFS % (95%CI)^c^	0.57 (0.37–0.73)	0.44 (0.20–0.66)	0.622
3-year OS % (95%CI)^c^	0.41 (0.19–0.63)	0.54 (0.25–0.76)	0.774

^a^percentages may not add to 100% due to missing values of defined variables, missing clinicopathological information was assumed to be missing at random. Variables are indicated as absolute numbers, %, median or range. Age, RFS and OS were evaluated using the Kaplan-Meier method. FIGO stage, residual disease, numbers of chemotherapy cycles and chemoresistance were analyzed using the Chi-Square or the Fisher’s Exact test

^b^*CS* chemosensitive, *CR* chemoresistant

^c^*RFS* recurrence-free survival, *OS* overall survival

Regarding the OX40 immune cell density (high vs. low), neither in primary nor recurrent cancer a significant association with clinicopathological features like FIGO stage, residual disease, numbers of chemotherapy cycles was shown. Also no correlation with RFS or OS was found (Tables 2 and 3).

### OX40 positive tumor expression in primary and recurrent OC

The mean number of positive OX40 tumor expression in primary and recurrent cancer biopsies was 214.0 (±28.6 SE) and 231.4 (±57.9 SE), respectively.

**Fig. 2:**
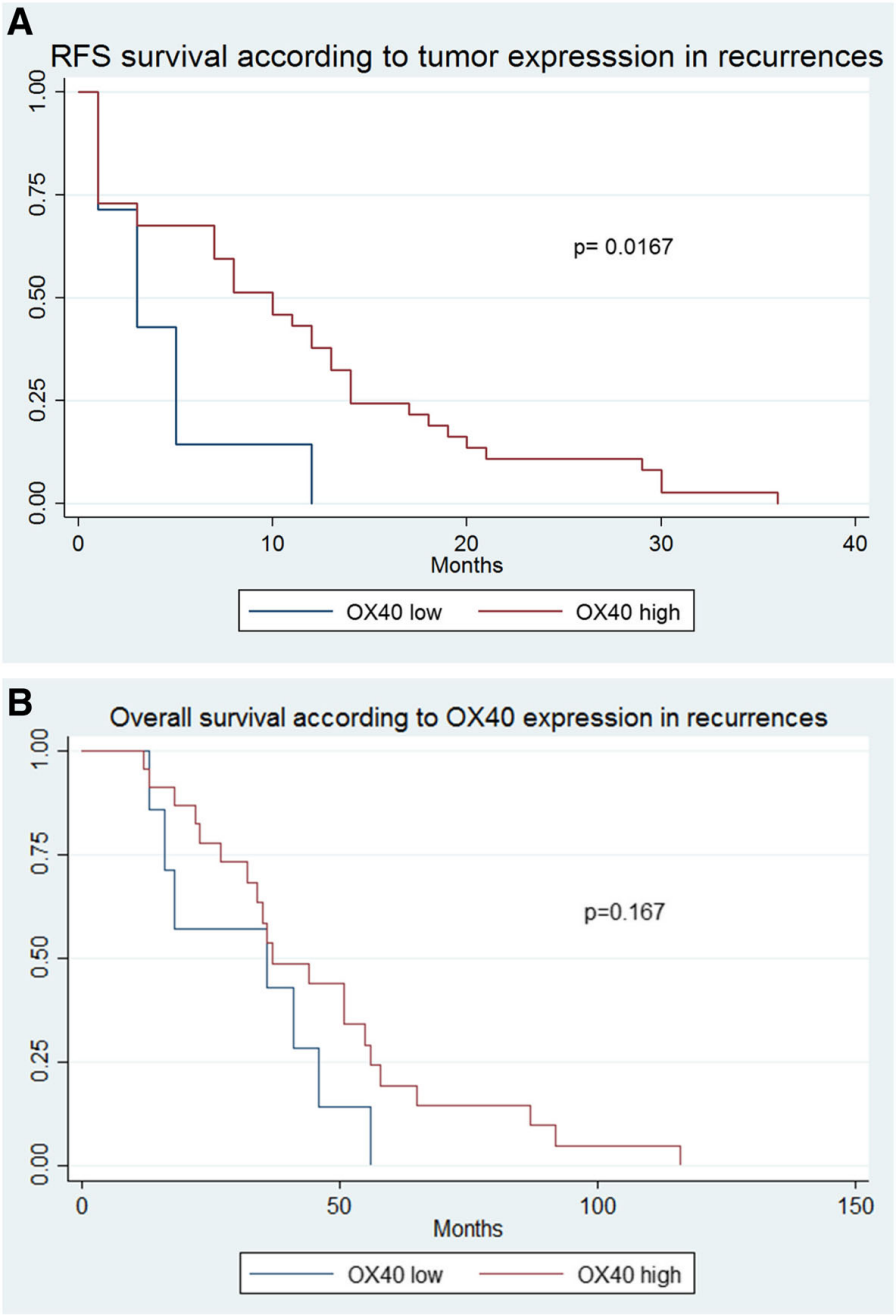
**a** Kaplan Meier survival curve of recurrence-free survival according to OX40 expression in recurrent cancer biopsies, Impact of OX40+ expression by tumor cells in recurrent cancer biopsies on recurrence-free survival in patients with high grade ovarian carcinoma. Kaplan-Meier recurrence-free survival curve was split according to OX40+ expression in patients bearing high grade ovarian carcinoma as indicated. Cut-off value established by regression tree analysis was 12.5 cells/punch. Blue line indicates to tumors with low OX40+ expression and red line refers to tumors with high OX40+ expression. **b** Impact of OX40+ expression by tumor cells in recurrent cancer biopsies on overall survival in patients with high grade ovarian carcinoma. Kaplan-Meier overall survival curve was split according to OX40+ expression in patients bearing high grade ovarian carcinoma as indicated. Cut-off value established by regression tree analysis was 12.5 cells/punch. Blue line indicates to tumors with low OX40+ expression and red line refers to tumors with high OX40+ expression.

**Fig. 3:**
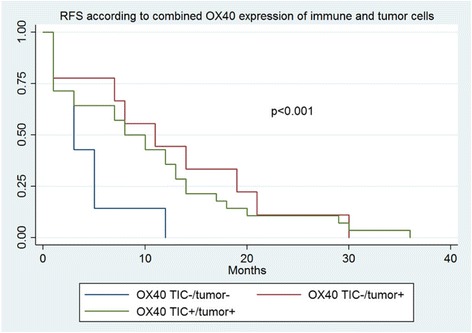
Kaplan Meier survival curve of recurrence-free survival according to OX40 expression of immune and tumor cells.

Patients with high OX40 expression in tumor cells in recurrent cancer biopsies showed a significantly increased chemosensitivity (*p* = 0.008) and improved 6-month RFS compared to patients with a low count of OX40 positive tumor cells (*p =* 0.017) (Table 4 and Fig. 2a). In contrast, OS did not significantly differ between the two groups (Fig. 2b). Additionally, no significant association was observed regarding response to chemotherapy in primary OC (Table 5). When combining OX40 positive and negativ immune and tumor cells, a significant worse RFS for the group with negative immune cells as well as negative tumor cells was found (Fig. 3).

**Table 4:** Patients’ characteristics according to dichotomized distribution of OX40 expression by tumor cells in recurrent cancer biopsies in the overall cohort (cut-off = 12.5; *n* = 44)^a^

	OX40 high	OX40 low	*p*-value
	*n* = 37 (100%)	*n* = 7 (100%)	
Age (median, range)	57 (34–77)	63 (41–76)	0.386
FIGO stage			
II	1 (2.7)	0	0.777
IIIA	1 (2.7)	0	
IIIB	5 (13.5)	0	
IIIC	24 (64.9)	5 (71.4)	
IV	6 (16.2)	2 (28.6)	
Residual disease			
None	13 (35.1)	2 (28.6)	0.406
< 2 cm	15 (40.5)	1 (14.3)	
> 2 cm	9 (24.3)	3 (42.9)	
Numbers of chemotherapy cycles			
< 6	7 (18.9)	0	0.244
6 or more	30 (81.0)	6 (85.7)	
CS^b^	29 (78.4)	2 (28.6)	0.008
CR^b^	8 (21.6)	5 (71.4)	
6-month RFS % (95%CI)^c^	0.59 (0.42–0.73)	0.14 (0.01–0.46)	0.017
3-year OS % (95%CI)^c^	0.48 (0.27–0.66)	0.43 (0.10–0.73)	0.167

^a^percentages may not add to 100% due to missing values of defined variables, missing clinicopathological information was assumed to be missing at random. Variables are indicated as absolute numbers, %, median or range. Age, RFS and OS were evaluated using the Kaplan-Meier method. FIGO stage, residual disease, numbers of chemotherapy cycles and chemoresistance were analyzed using the Chi-Square or the Fisher’s Exact test

^b^*CS* chemosensitive, *CR* chemoresistant

^c^*RFS* recurrence-free survival, *OS* overall survival

**Table 5:** Patients’ characteristics according to dichotomized distribution of OX40 expression by tumor cells in primary cancer biopsies in the overall cohort (cut-off = 144.5; *n* = 47)^a^

	OX40 high	OX40 low	*p*-value
	*n* = 27 (100%)	*n* = 20 (100%)	
Age (median, range)	58 (34–73)	64 (39–77)	0.197
FIGO stage			
II	1 (3.7)	0	0.643
IIIA	0	1 (5.0)	
IIIB	4 (14.8)	1 (5.0)	
IIIC	18 (66.7)	14 (70.0)	
IV	4 (14.8)	4 (20.0)	
Residual disease			
None	11 (40.7)	5 (25.0)	0.277
< 2 cm	10 (37.0)	7 (35.0)	
> 2 cm	5 (18.5)	8 (40.0)	
Numbers of chemotherapy cycles			
< 6	3 (11.1)	4 (20.0)	0.428
6 or more	23 (85.2)	16 (80.0)	
CS^b^	20 (74.1)	13 (65.0)	0.501
CR^b^	7 (25.9)	7 (35.0)	
6-month RFS % (95%CI)^c^	0.56 (0.35–0.72)	0.50 (0.27–0.69)	0.472
3-year OS % (95%CI)^c^	0.43 (0.22–0.62)	0.45 (0.17–0.71)	0.869

^a^percentages may not add to 100% due to missing values of defined variables, missing clinicopathological information was assumed to be missing at random. Variables are indicated as absolute numbers, %, median or range. Age, RFS and OS were evaluated using the Kaplan-Meier method. FIGO stage, residual disease, numbers of chemotherapy cycles and chemoresistance were analyzed using the Chi-Square or the Fisher’s Exact test

^b^*CS* chemosensitive, *CR* chemoresistant

^c^*RFS* recurrence-free survival, *OS* overall survival

### OX40 positive immune cell infiltration and tumor expression by chemosensitivity

The number of OX40 positive tumor cells in the chemosensitive and chemoresistant group were 225.2 (±36.7 SE) vs 187.8 (±43.1 SE) (*p* = 0.585) in primary cancer biopsies and 266.8 (±76.5 SE) vs 147.0 (±49.5 SE) (*p* = 0.126) in recurrent cancer biopsies. The corresponding values for OX40 positive immune cells were 30.0 (±6.0 SE) vs 15.3 (±3.7 SE) (*p* = 0.149) and 31.7 (±11.5 SE) vs 11.2 (±3.8 SE) (*p* = 0.126), respectively.

### Correlation analysis of OX40 positive immune cell infiltration and tumor expression

Further we performed a correlation analysis. There was significant correlation between OX40 positive tumor cells and TICI in primary OC (rho = 0.662; *p* < 0.001) as well as in recurrent OC (rho = 0.658; *p* < 0.001). No correlation was found for OX40 positive tumor cells in primary or recurrent OC (rho = 0.277; *p* = 0.069). On the other hand, the correlation between TICI in primary and recurrent OC was statistically significant (rho = 0.543; *p* < 0.001). However, there was no synergistic effect combining the two scores as RFS was not influenced (data not shown).

### Uni- and multivariate cox regression survival analysis

In univariate cox regression survival analysis, high density of OX40 positive tumor cells in recurrent cancer biopsies was significantly associated with longer RFS (HR 0.39, 95%CI 0.16–0.94, *p* = 0.036) (Table 6). Additionally, residual disease of more than 2 cm and the number of chemotherapy cycles received also showed a significant correlation with RFS (HR 3.67, 95%CI 1.62–8.31 (*p* = 0.002) and HR 1.28, 95%CI 1.05–1.55; (*p* = 0.013), respectively). Lastly, in multivariate analysis no significant correlation to RFS was found regarding patient’s age, residual disease, FIGO classification and number of chemotherapy cycles (Table 6).

**Table 6:** Uni- and multivariate Hazard Cox regression analysis of recurrence-free survival considering the dichotomized tumor expression in recurrent cancer biopsies

	Univariate			Multivariate		
	HR	95% CI	*p*-values	HR	95% CI	*p*-values
Age	1.00	0.97 - 1.03	0.888	1.00	0.97 - 1.03	0.819
OX40high vs OX40low	0.39	0.16 - 0.94	**0.036**	0.74	0.18 - 3.01	0.676
Residual disease < 2 cm	1.13	0.56 - 2.28	0.724	0.88	0.40 - 1.91	0.747
Residual disease > 2 cm	3.67	1.62 - 8.31	**0.002**	2.78	1.09 - 7.06	**0.032**
No. of chemotherapy cycles	1.28	1.05 - 1.55	**0.013**	1.12	0.82 - 1.53	0.477
FIGO IIIA	0.34	0.02 - 5.72	0.455	0.43	0.02 - 8.31	0.573
FIGO IIIB	0.93	0.11 - 8.04	0.944	0.90	0.10 - 8.17	0.928
FIGO IIIC	1.21	0.16 - 9.03	0.851	1.02	0.13 - 8.24	0.985
FIGO IV	1.48	0.18 - 11.94	0.712	0.99	0.11 - 9.00	0.991

Multivariate analyses showing Hazard Ratios and p-value for all recurrent cancer biopsies (*n* = 43 less than 44 due to missing value) conferred by categorized OX40 expression, age, residual disease after cytoreductive surgery, number of chemotherapy cycles and FIGO classification

### Correlation analysis of OX40 and IL-17 positive tumor immune cell infiltration

In order to investigate a possible association between OX40 and IL-17 positive tumor immune cell infiltration, a correlation analysis was performed. OX40 positive immune cells in recurrent carcinomas correlated significantly with IL-17 positive immune cells in primary carcinomas (r_s_ = 0.34; *p* = 0.023). No other significant correlation was found between OX40 and IL-17 expression (Table 7).

**Table 7:** Correlation analysis of OX40 and IL-17 positive tumor immune cell infiltration

	IL-17+ TICI in primary^a^		IL-17 + TICI in recurrent^b^
OX40+ TICI in primary^c^	***rs***	0.176	0.002
	***p***	0.254	0.989
OX40+ TICI in recurrent^d^	***rs***	0.341	0.245
	***p***	**0.023**	0.109
OX40+ tumor in primary^e^	***rs***	0.096	0.049
	***p***	0.537	0.755
OX40+ tumor in recurrent^f^	***rs***	0.276	0.205
	***p***	0.070	0.182

Correlation analysis of OX40 and IL-17 showing rho and *p*-value

^a^IL-17 positive immune cells in primary cancer biopsies

^b^IL-17 positive immune cells in recurrent cancer biopsies

^c^OX40 positive immune cells in primary cancer biopsies

^d^OX40 positive immune cells in recurrent cancer biopsies

^e^OX40 expression by tumor cells in primary cancer biopsies

^f^ OX40 expression by tumor cells in recurrent cancer biopsies

## Discussion

Surgical debulking followed by platinum-based chemotherapy is the standard treatment for OC. High-grade serous carcinomas are characterized by initial good response to chemotherapy with subsequent acquisition of increasing resistance at each recurrence [4, 40, 41]. Current second-line therapies are generally not curative, resulting in short term progression-free survival for most patients [41].

As stated, it would be helpful to have predictive biomarkers for chemoresistance and -sensitivity for an individualized therapy regimen. Additional survival benefit could be achieved by extended chemotherapy or repetitive surgical procedures depending on the assumed response to chemotherapy. We therefore examined the indicative value of OX40 for chemosensitivity in OC.

As previously mentioned the occurrence of tumor-infiltrating lymphocytes delay tumor progression through several mechanisms and is associated with survival benefits in a variety of patients with different tumors [10, 42]. Furthermore, in advanced ovarian cancer intratumoral T cells has been shown to correlate with improved clinical outcome [9, 10]. We could show that primary tumors with a high incidence of OX40 immune cell infiltration were significantly more often chemosensitive. Accordingly, they showed a delayed tumor recurrence, at earliest 6 months after completion of platinum-based chemotherapy.

Most importantly, our study shows that patients with a higher incidence of OX40 receptors expressed by the tumor cells have an improved RFS and show increased chemosensitivity in their recurrent cancer.

In line with this study, experimental models show that OX40 co-stimulatory molecule enhanced CD4+ and CD8+ cell proliferation, stimulated cytokine production, and increased survival of antigen-specific memory T cell [19–21].

OX40 targeted immunotherapy treatment has already been tested in a clinical trial for a variety of cancers. They could show tumor regression after just one cycle of mouse monoclonal antibody that agonizes human OX40 signaling in patients with advanced cancer [24].

The results of our study underline the importance of OX40 expression in recurrent OC tissue for prolonged recurrent free survival.

In our previous study, we identified IL-17 positive TICI as a predictive marker for chemosensitivity in primary and recurrent OC [25]. Interestingly, in the current study, OX40 positive TICI in recurrent carcinomas significantly correlated with IL-17 positive TICI in primary carcinomas. Additional larger studies are necessary to achieve an in-depth understanding of the interaction between OX40 and IL-17 and response to chemotherapy in OC.

## Conclusion

Based on our results, OX40 receptor expression seems to be a valid marker for a personalized treatment concerning a second line therapy for patients with ovarian cancer. We suggest that patients with a high OX40 tumor cell expression in recurrent carcinoma might benefit from repeated chemotherapy.

## Abbreviations

APC: Antigen-presenting cells
CI: Confidence intervals
HR: Hazard ratios
IHC: Immunohistochemistry
MPO: Myeloperoxidase
OC: Ovarian carcinoma
OS: Overall survival
RFS: Recurrent free survival
TICI: Tumor immune cell infiltration
TMA: Tissue microarrays
